# Identification and quantification of protein *S*-nitrosation by nitrite in the mouse heart during ischemia

**DOI:** 10.1074/jbc.M117.798744

**Published:** 2017-07-14

**Authors:** Edward T. Chouchani, Andrew M. James, Carmen Methner, Victoria R. Pell, Tracy A. Prime, Brian K. Erickson, Marleen Forkink, Gigi Y. Lau, Thomas P. Bright, Katja E. Menger, Ian M. Fearnley, Thomas Krieg, Michael P. Murphy

**Affiliations:** From the ‡ Department of Cancer Biology, Dana–Farber Cancer Institute, Boston, Massachusetts 02284-9168,; the § Department of Cell Biology, Harvard Medical School, Boston, Massachusetts 02115,; the ¶ Medical Research Council Mitochondrial Biology Unit, University of Cambridge, Cambridge Biomedical Campus, Cambridge CB2 0XY, United Kingdom, and; the ‖ Department of Medicine, University of Cambridge, Addenbrooke's Hospital, Hills Road, Cambridge CB2 2QQ, United Kingdom

**Keywords:** heart, ischemia, mitochondria, proteomics, redox regulation, OxICAT, S-nitrosation, nitrite, redox

## Abstract

Nitrate (NO_3_^−^) and nitrite (NO_2_^−^) are known to be cardioprotective and to alter energy metabolism *in vivo*. NO_3_^−^ action results from its conversion to NO_2_^−^ by salivary bacteria, but the mechanism(s) by which NO_2_^−^ affects metabolism remains obscure. NO_2_^−^ may act by *S*-nitrosating protein thiols, thereby altering protein activity. But how this occurs, and the functional importance of *S*-nitrosation sites across the mammalian proteome, remain largely uncharacterized. Here we analyzed protein thiols within mouse hearts *in vivo* using quantitative proteomics to determine *S*-nitrosation site occupancy. We extended the thiol-redox proteomic technique, isotope-coded affinity tag labeling, to quantify the extent of NO_2_^−^-dependent *S*-nitrosation of proteins thiols *in vivo*. Using this approach, called SNOxICAT (*S*-nitrosothiol redox isotope-coded affinity tag), we found that exposure to NO_2_^−^ under normoxic conditions or exposure to ischemia alone results in minimal *S*-nitrosation of protein thiols. However, exposure to NO_2_^−^ in conjunction with ischemia led to extensive *S*-nitrosation of protein thiols across all cellular compartments. Several mitochondrial protein thiols exposed to the mitochondrial matrix were selectively *S*-nitrosated under these conditions, potentially contributing to the beneficial effects of NO_2_^−^ on mitochondrial metabolism. The permeability of the mitochondrial inner membrane to HNO_2_, but not to NO_2_^−^, combined with the lack of *S*-nitrosation during anoxia alone or by NO_2_^−^ during normoxia places constraints on how *S*-nitrosation occurs *in vivo* and on its mechanisms of cardioprotection and modulation of energy metabolism. Quantifying *S*-nitrosated protein thiols now allows determination of modified cysteines across the proteome and identification of those most likely responsible for the functional consequences of NO_2_^−^ exposure.

## Introduction

**Figure 1.**
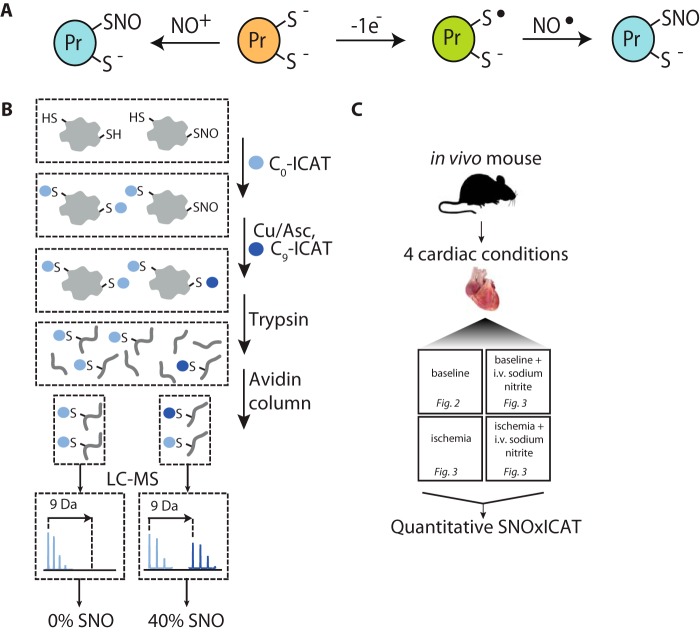
**The SNOxICAT methodology.** *A*, *S*-nitrosation of sensitive surface protein thiols. A small subset of mitochondrial surface protein thiols are sensitive to *S*-nitrosation, which is thought to occur by two mechanisms. The thiolate form of a sensitive thiol can react with a nitrosonium ion to form an S-nitrosothiol (*left*). Alternatively, the thiyl radical form, generated by one electron oxidation of a thiol or thiolate, may react directly with NO^•^ to form an *S*-nitrosothiol (*right*). The resulting reversible modification may have functional consequences for the target protein or, alternatively, could block other irreversible oxidative modifications from occurring on the thiol. *B*, SNOxICAT methodology. Following *in vivo* interventions, mouse myocardial tissue is excised, and thiols are stabilized by homogenization in 20% TCA containing 0.5% sulfanilamide. Unmodified protein thiols are labeled with C_0_-ICAT, and then *S*-nitrosated thiols are chemically reduced and labeled with C_9_-ICAT, allowing for identification and quantification of the *S*-nitrosation status of protein thiols across the proteome. The peptides are then isolated and analyzed by mass spectrometry. *C*, experimental design for comparative analysis of *S*-nitrosation status in the mouse heart.

Until recently, nitrate (NO_3_^−^) and nitrite (NO_2_^−^) were considered biologically inert byproducts of NO metabolism in mammals, but now it is clear that they impact significantly on mammalian physiology and energy metabolism (1). Dietary NO_3_^−^ is converted to NO_2_^−^ by salivary bacteria (2), and this NO_2_^−^ is thought to account for most of the biological properties of NO_3_^−^ in mammals. For example, NO_2_^−^ can act *in vivo* as a cytoprotectant during cardiac ischemia–reperfusion injury (3–5) and as a modulator of mitochondrial function (4) and exercise efficiency (6). How NO_2_^−^ exerts its effects *in vivo* is unclear, but a major component seems to be the *S*-nitrosation (or *S*-nitrosylation) of cysteine residues on proteins (7) (Fig. 1*A*). Protein cysteine *S*-nitrosation is a posttranslational modification through which NO and its metabolites exert some of their actions by reversibly altering protein function (8–18). Therefore, a plausible mode of action of NO_2_^−^ *in vivo* is via protein *S*-nitrosation (Fig. 1*A*). This notion is supported by a number of studies that have shown that NO_2_^−^ treatment *in vivo* broadly increases protein *S*-nitrosation (3–5).

Even so, the functional relevance of *S*-nitrosation by NO_2_^−^ *in vivo* remains uncertain. This is in part because the proteomic methods used to identify the cysteine residues and proteins affected by *S-*nitrosation (18–20) do not measure the extent of cysteine modification. Quantification of the extent of protein *S*-nitrosation on a particular cysteine residue (*i.e.* site occupancy) is essential to identify the functional nodes through which *S*-nitrosation may exert its physiological and pharmacological roles. Although many cysteine residues may be modified indiscriminately to a low extent, these alterations are unlikely to have major physiological consequences; instead, we expect that only cysteine residues that are extensively modified by NO_2_^−^ can explain the biological actions of this signaling modality (although we cannot exclude gain-of-function effects). To address this unmet need, we adapted cysteine-reactive isotope-coded affinity tag (ICAT)^4^ approaches (21), which both identify and quantify the extent of reversible modification of a cysteine residue (22–25). To do this, we modified sample processing so that, instead of identifying all reversibly modified protein thiols, only *S*-nitrosated cysteines are analyzed and quantified. Application of this approach, which we term SNOxICAT, allowed us to identify cysteine residues that are *S*-nitrosated and, most importantly, quantify the extent of their *S*-nitrosation.

Here we have used SNOxICAT to study the impact of NO_2_^−^ on the mouse heart *in vivo*. The heart was chosen because of the substantial interest in uncovering the mechanisms underlying both the cytoprotective and other metabolic modulatory effects of NO_2_^−^ on cardiac function (1). Using this approach, we have identified a range of protein thiols that were *S*-nitrosated by NO_2_^−^ in the heart and quantified the extent of this modification. Interestingly, the number of protein thiols in the heart substantially modified by NO_2_^−^ was dramatically increased by ischemia, suggesting that the combination of NO_2_^−^ and ischemia is necessary for extensive *S*-nitrosation *in vivo. S*-nitrosation was not increased by NO_2_^−^ during normoxia or by ischemia alone. Furthermore, several protein thiols *S*-nitrosated by NO_2_^−^ during ischemia were on mitochondrial proteins, providing a potential mechanistic link to the effects of NO_2_^−^ on mitochondrial function.

## Results and discussion

### Using SNOxICAT to identify and quantify S-nitrosation of cardiac proteins in vivo

In exploring how NO_2_^−^ might lead to protein *S*-nitrosation, and to infer proteins with potential physiological roles, we wanted to identify the sites of *S*-nitrosation *in vivo* and also quantify the extent of modification on individual targets. However, *S*-nitrosothiols are too unstable to survive analysis; hence, they need to be measured indirectly. To do this, we extended the redox-proteomic method OxICAT, which uses Cys-ICAT chemistry to both quantify and identify thiols that are reversibly oxidized, which has been applied to *Escherichia coli* (22), *Saccharomyces cerevisiae* (25), *Caenorhabditis elegans* (23), *Drosophila melanogaster* (24), and to the mouse heart (26). OxICAT identifies reversibly oxidized protein thiols, so we refined OxICAT to develop the SNOxICAT approach, which reports on *S*-nitrosated protein thiols (Fig. 1*B*). To do this, tissue samples were rapidly stabilized by solubilization in TCA in the presence of sulfanilamide (Fig. 1*B*), which both preserved endogenous *S-*nitrosothiols and prevented artifactual *S*-nitrosation of free thiols by acidified NO_2_^−^ during sample workup (27) (Fig. 1*B*). Following stabilization, the samples were neutralized, and unmodified cysteine residues were labeled with “light” C_0_-ICAT (Fig. 1*B*), and then protein *S*-nitrosothiols were labeled with “heavy” C_9_-ICAT after their selective reduction by Cu(I) was achieved by adding low concentrations of ascorbic acid and CuSO_4_ (Fig. 1*B*) (28, 29). Then the sample was digested with trypsin, and cysteine-containing peptides that had reacted with C_0_-ICAT or C_9_-ICAT were enriched by affinity purification using the biotin tag on the ICAT reagents, separated by liquid chromatography, and analyzed by MS. As both the C_0_ and C_9_ ICAT labeled peptides are chemically identical and elute at the same point after liquid chromatography, this enabled the extent of modification of the cysteine residue of interest to be quantified. In-line sequencing enabled the peptides and their cognate proteins to be identified by MS-MS. Hence, we could identify protein cysteine residues that were *S*-nitrosated *in vivo* and, at the same time, determine the extent of this modification.

Quantitative MS-MS data obtained from this approach were applied to several cardiac interventions *in vivo* (Fig. 1*C*). Data from individual experiments were cross-referenced using an HPLC-MS alignment algorithm to maximize peptide coverage (30). This enabled both the identification and the quantification of heavy and light ICAT signals for 2203 cysteine residues in the myocardium that came from 721 proteins (supplemental Table S1). These signals were converted to a percent *S*-nitrosation value ((100 × heavyICAT)/(heavyICAT + lightICAT)) for each particular cysteine residue (supplemental Table S2). From the total pool of identified cysteine peptides with quantitative *S*-nitrosation data, between 1235 and 1868 were quantified at least three times. One complication with quantifying *S*-nitrosation is that many cysteines are not *S-*nitrosated at all; hence, ∼55% of residues only have one ICAT label. A failure to observe both labels can occur because the residue is either not *S-*nitrosated or is fully *S-*nitrosated; alternatively, it may occur for technical reasons related to MS instrumentation and software. Thus, although it is biologically important to include these 0 and 100% *S*-nitrosated values, a proportion of them are technical artifacts related to a failure in peak identification. To provide an accurate representation of *in vivo S*-nitrosation required differentiation between these two scenarios. To do this, we noted that a hallmark of informative data points is that they should have similar *S*-nitrosation across biological replicates and, thus, a low corresponding S.E. Conversely, apparent *S*-nitrosation values arising because of technical artifacts are unconstrained by the *S*-nitrosation of their biological replicates and, thus, are likely to exhibit higher S.E. values. Thus, a further curated dataset was generated where peptides with a highly variable cysteine *S*-nitrosation state were assumed to arise because of technical artifact and were excluded (see supplemental Fig. S1 and “Experimental procedures” for further discussion), leaving 866–1581 peptides (supplemental Table S3). The total number of cysteine-containing peptides in an *in silico* trypsin digest of the total mouse genome is ∼320,000; hence, we observed about ∼0.5% of the total number of cysteine residues present in the mouse genome.

**Figure 2.**
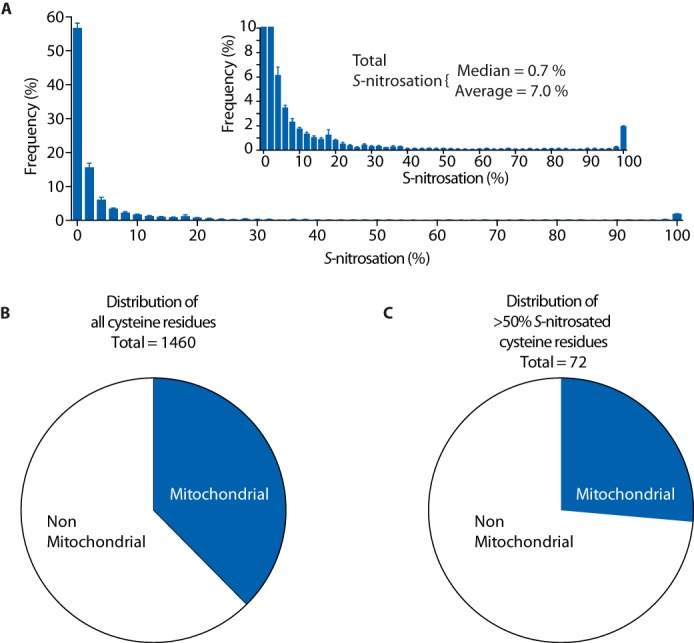
**Quantification of basal *S*-nitrosation in the mouse heart by SNOxICAT.** *A*, overall detailed distribution of percentage *S*-nitrosation status of protein thiols in the heart under baseline conditions. The percentage *S*-nitrosation status is distributed in 2% quantiles, indicating the proportion of total cysteines identified exhibiting a particular level of *S*-nitrosation. The *inset* shows an expansion of these findings. *B* and *C*, pie charts showing the relative amount of mitochondrial and non-mitochondrial *S*-nitrosated cysteine residues in the myocardium under basal conditions for all *S*-nitrosated proteins detected (*B*) and for proteins that were *S*-nitrosated by more than 50% (*C*).

We first used this approach to explore the baseline levels of *S*-nitrosation in the normoxic heart (Fig. 2*A* and supplemental Table S1). Under normoxic conditions, we found 907 cysteine residues with some degree of sensitivity to copper/ascorbate, consistent with *S*-nitrosation. This is in agreement with previous studies that have established widespread *S*-nitrosation under baseline conditions *in vivo* (19). However, of all the cysteine residues identified, over half were less than 1% *S*-nitrosated (Fig. 2*A*), whereas only a small number (∼5%, *n* = 72) were greater than 50% *S*-nitrosated. Thus, under basal conditions, there is low-level *S*-nitrosation of several protein thiols, but very few cysteine residues are extensively modified, demonstrating the importance of quantifying site occupancy.

We were particularly interested in assessing the degree of protein *S*-nitrosation in mitochondria compared with the rest of the cell. Therefore, we divided *S*-nitrosated proteins into mitochondrial or non-mitochondrial (Fig. 2*B*) and then further separated proteins that were *S*-nitrosated by greater than 50% (Fig. 2*C*). These analyses showed that there was a slight diminishment in enrichment of *S*-nitrosation in mitochondrial proteins under basal conditions.

### Effect of NO_2_^−^ on protein S-nitrosation in the normoxic and ischemic heart

**Figure 3.**
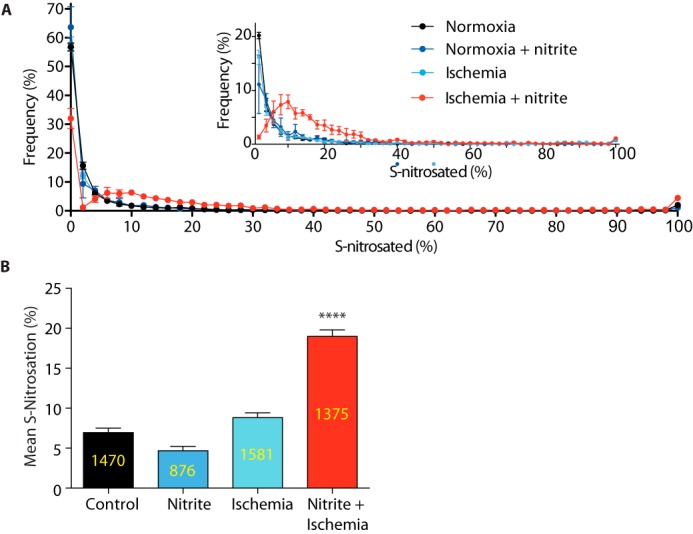
**Effect of NO_2_^−^ on the *S*-nitrosothiol proteome in the mouse heart under normoxic and ischemic conditions.** *A*, distribution of percentage *S*-nitrosation status of protein thiols in the heart under normoxia, normoxia + NO_2_^−^, ischemia and ischemia + NO_2_^−^. The percentage *S*-nitrosation status is distributed in 2% quantiles, indicating the proportion of total cysteines identified exhibiting a particular level of *S*-nitrosation. The *inset* shows an expansion of these findings. *B*, the mean percentage *S*-nitrosation of protein thiols under the four conditions. Data are means ± S.E. The numbers of peptides analyzed are indicated within each column. ****, *p* < 0.001 relative to control.

To assess the effect of NO_2_^−^ on protein thiols in the heart, we injected NaNO_2_ intravenously into mice at a concentration that has been reported to protect the heart against ischemia–reperfusion injury and drive *S*-nitrosation in the heart (4, 31) (Fig. 1*C*). The *S*-nitrosation status of cysteine residues in the heart of normoxic mice was largely unchanged by NO_2_^−^ injection (Fig. 3*A*). As the extent of *S*-nitrosation by NO_2_^−^ has been shown to increase upon ischemia (4), we next set out to assess the effects of NO_2_^−^ on cardiac *S*-nitrosation following exposure to 25 min of ischemia (Fig. 3*A*). Under these conditions, administration of NO_2_^−^ led to a dramatic increase in overall *S*-nitrosation in the ischemic myocardium, although ischemia alone did not increase *S*-nitrosation (Fig. 3*A*). These changes are quantified in Fig. 3*B* as the means of the extent of *S*-nitrosation of all the peptides. Together, the data in Fig. 3, *A* and *B*, suggest that there was an increase in the average extent of *S*-nitrosation when there was a combination of NO_2_^−^ and ischemia but not with either alone.

**Figure 4.**
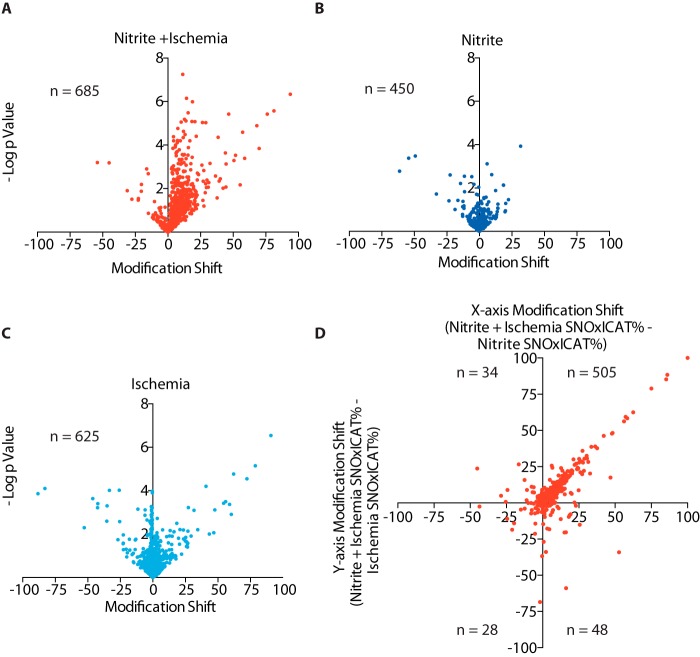
**Relative effect of NO_2_^−^ on the *S*-nitrosation of individual peptides in the mouse heart under normoxic and ischemic conditions.** *A–C*, volcano plots of the *p* value for the change in *S*-nitrosation for individual peptides, comparing the indicated conditions against normoxic control (Δ% points = % *S*-nitrosation under condition − % *S*-nitrosation under normoxia). The *n* values show the number of peptides for which there was sufficient data under the indicated condition and normoxia. *A*, NO_2_^−^ and ischemia. *B*, NO_2_^−^ alone. *C*, ischemia alone. *D*, comparison of the shift in in *S*-nitrosation for a given peptide upon going from ischemia alone to ischemia + NO_2_^−^, shown on the *y* axis, plotted against the shift in *S*-nitrosation for a given peptide upon going from NO_2_^−^ alone to ischemia + NO_2_^−^.

To test the effectiveness of the dataset curation and further assess changes in *S*-nitrosation, we switched our focus from aggregate levels of *S*-nitrosation under different conditions to the *S*-nitrosation of individual cysteine residues. As there was no apparent biological shift in *S-*nitrosation at the population level in response to NO_2_^−^ alone relative to the control, this provided an opportunity to test the validity of our curation described previously. As expected, the raw and curated plots of these two independently determined values coalesced around a line of slope ∼1, with most values clustering near the origin (supplemental Fig. S1, *A* and *C*). Furthermore, the shift in *S-*nitrosation with NO_2_^−^ and ischemia is readily apparent in both the non-curated and curated datasets (supplemental Fig. S1, *B* and *D*). The data points excluded from analysis show no such correlation and lacked an excess of statistically interesting comparisons above the false discovery rate (supplemental Fig. S1, *E* and *F*). Together, this argues that our curation is appropriate and effective. Next, we compared how the extent of *S*-nitrosation of individual peptides is altered under different conditions. Volcano plots were created to assess changes in *S*-nitrosation on individual peptides in response to NO_2_^−^ alone, ischemia alone, and ischemia + NO_2_^−^, all relative to control conditions. There was a clear bias toward peptides being significantly *S*-nitrosated by ischemia + NO_2_^−^ (Fig. 4*A*). This was not observed in the symmetrical plots for NO_2_^−^ (Fig. 4*B*) or ischemia alone (Fig. 4*C*). This is illustrated further in Fig. 4*D*, which examines the increase in *S*-nitrosation on individual peptides. Along the *x* axis, we plotted the increase in *S*-nitrosation on peptides when tissue exposed to NO_2_^−^ and ischemia is compared with normoxic tissue exposed to NO_2_^−^. Along the *y* axis, we plotted the increase in *S*-nitrosation on peptides when tissue exposed to NO_2_^−^ and ischemia is compared with ischemic tissue. The majority of peptides (82%) appear in the top right quadrant with a slope of ∼1, consistent with *S*-nitrosation being a process that depends on both ischemia and NO_2_^−^. Of the 1081 cysteines present in both control tissue and that exposed to NO_2_^−^ and ischemia, statistical comparisons could be made for 644, and of these, 264 were significantly changed (*p* < 0.05). This is an excess of 232 over the false discovery rate of 32 (supplemental Fig. S1*D*). After Benjamini-Hochberg correction for multiple comparisons, 102 cysteine residues could be identified as changing in response to NO_2_^−^ and ischemia (supplemental Table S4). However, although there is a statistically significant shift in the *S-*nitrosation status of a large number of peptides with ischemia + NO_2_^−^, many cysteines are not *S*-nitrosated at all in the control heart and are consequently lost from statistical comparisons. Consequently, we have focused on large absolute shifts in *S*-nitrosation, as these are most likely to contribute to biologically meaningful changes to protein function within the tissue. Supplemental Table S4 shows the 163 sites where NO_2_^−^ in the ischemic myocardium increases *S*-nitrosation by more than 10 percentage points above conditions of normoxia, NO_2_^−^ alone, or ischemia alone.

Thus, we conclude that there is a substantial population of protein cysteines that become selectively *S*-nitrosated by NO_2_^−^ in the ischemic heart but that are unmodified by NO_2_^−^ under normoxic conditions or by ischemia alone. This is in agreement with previous studies that show that overall levels of *S*-nitrosothiols increase following NO_2_^−^ administration under ischemic conditions (32).

### S-nitrosation of mitochondrial oxidoreductases by NO_2_^−^ during ischemia

**Figure 5.**
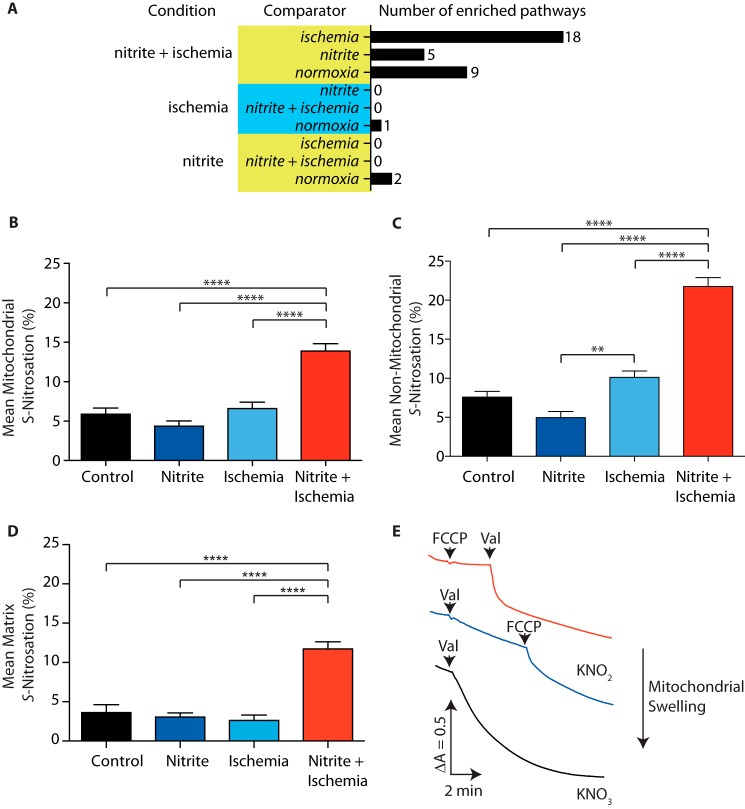
**Intracellular localization of *S*-nitrosated protein thiols upon NO_2_^−^ and ischemia.** *A*, pathway analysis of proteins containing cysteine residues sensitive to selective *S*-nitrosation (>10 percentage point average shift in *S*-nitrosation) in each treatment protocol, indicating the number of significantly enriched pathways. *B* and *C*, subcellular localization of cysteines selectively targeted for *S*-nitrosation by NO_2_^−^ under ischemic conditions. *D*, TCA cycle targets of *S*-nitrosation. *E*, NO_2_^−^ uptake assessed by swelling of mitochondria. Mitochondria (0.5 mg protein/ml) were incubated in 10 mm Tris-HCl (pH 7.2) at 37 °C supplemented with rotenone (4 μg/ml) and containing either KNO_2_ or KNO_3_ (100 mm). Absorbance was measured at 550 nm, and valinomycin (1 μm) or FCCP (2 μm) was added where indicated. Shown is a typical result of three replicates. **, *p* < 0.01; ****, *p* < 0.001.

NO_2_^−^ has a number of effects on mitochondrial metabolism, and several of the most substantially modified targets of *S*-nitrosation were mitochondrial. To further examine the selective quantitative effects of *S*-nitrosation by NO_2_^−^ in the ischemic heart, we clustered proteins with cysteine thiols exhibiting a differential *S*-nitrosation status (defined as >10 percentage points between conditions) using gene ontology term enrichment, with the total identified population of cysteine thiol containing proteins as the reference background. This approach identified *S*-nitrosation targets specific to each intervention that clustered in specific cellular pathways (Fig. 5*A* and supplemental Table S5). Among the most prevalent targets selectively modified by ischemic NO_2_^−^ were mitochondrial oxidoreductases involved in redox metabolism, electron transport, and the mitochondrial tricarboxylic acid cycle pathways (Fig. 5*A* and supplemental Table S5). Interestingly, despite this selective *S*-nitrosation of mitochondrial oxidoreductases, the average extent of *S*-nitrosation of all mitochondrial peptides (Fig. 5*B*) was similar to that observed for non-mitochondrial peptides (Fig. 5*C*). This suggests that mitochondrial oxidoreductases may be selective targets for modification even when compared with other mitochondrial proteins. Indeed, many of the pathways identified as enriched targets in the ischemic NO_2_^−^ treatments are present in the mitochondrial matrix, as opposed to mitochondrial protein thiols that face the intermembrane space or are located on the outer membrane. The mitochondrial matrix is a distinct compartment from the rest of the cell, with an environment that may impact NO_2_^−^ metabolism during ischemia. To get a clearer picture of whether *S*-nitrosation was occurring in the mitochondrial matrix in response to exogenous NO_2_^−^, we limited the analysis to soluble enzymes from the TCA cycle, which are all present in the mitochondrial matrix (supplemental Table S3). When this was done, there was again a clear increase in *S*-nitrosation within the mitochondrial matrix in response to NO_2_^−^ and ischemia (Fig. 5*D*). Thus, the combination of NO_2_^−^ and ischemia leads to a clear increase in *S*-nitrosation within the mitochondrial matrix, although how this takes place is not known.

To explore how the combination of NO_2_^−^ and ischemia might impact the mitochondrial matrix, we examined whether NO_2_^−^ itself could gain access to the mitochondrial matrix. When mitochondria were incubated in a high concentration of KNO_3_, the organelles did not swell until the K^+^ ionophore valinomycin was added to enable K^+^ entry along with NO_3_^−^. Valinomycin allows for net uptake of KNO_3_ and, thereby, leads to swelling, consistent with the mitochondrial inner membrane being permeable to NO_3_^−^ (Fig. 5*E*). In contrast, when mitochondria were incubated in KNO_2_, addition of valinomycin did not lead to swelling (Fig. 5*E*), suggesting that the mitochondrial inner membrane is not permeable to NO_2_^−^ *per se*. However, when NO_2_^−^ uptake was examined in the presence of valinomycin, along with the uncoupler carbonyl cyanide *p*-trifluoromethoxyphenylhydrazone (FCCP) to render the inner membrane permeable to protons, it did lead to mitochondrial swelling (Fig. 5*E*). This indicates that, *in vivo*, NO_2_^−^ itself is unlikely to enter mitochondria but that its conjugate acid HNO_2_ (p*K_a_* = 3.16) can. Therefore, one possibility is that, *in vivo* during ischemia, NO_2_^−^ leads to mitochondrial protein *S*-nitrosation by generating membrane-permeant HNO_2_ that can diffuse into the matrix. This membrane permeability may be further enhanced by the acidic environment likely to be present in ischemic tissue and could contribute to the increase in *S-*nitrosation observed with NO_2_^−^ and ischemia relative to NO_2_^−^ alone. However, the mechanism by which NO_2_^−^ leads to *S*-nitrosation is unclear and may require the further reaction of HNO_2_ to potential *S*-nitrosating species such as N_2_O_3_ or catalysis by metalloenzymes.

**Figure 6.**
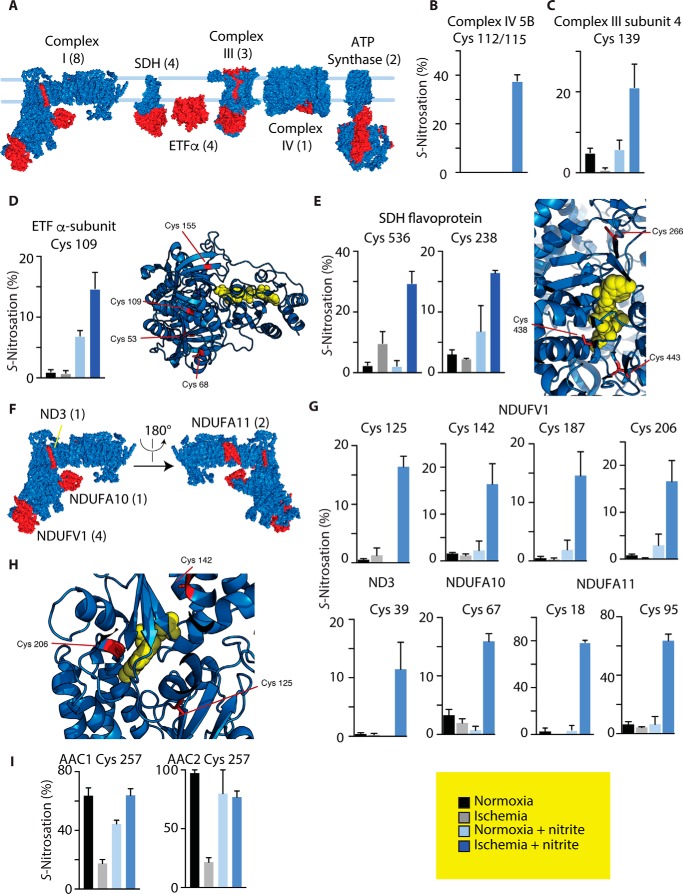
**Identification of mitochondrial protein cysteine and pathway targets of *S*-nitrosation caused by ischemic NO_2_^−^.** *A*, structures of OXPHOS enzymes, with subunits containing targets of ischemic *S*-nitrosation by NO_2_^−^ highlighted in *red* and the number of *S*-nitrosated cysteine residues given in *parentheses. B–E*, quantification of the *S*-nitrosation status of cysteine residues that are selectively modified by NO_2_^−^ during ischemia on complex IV (*B*), complex III (*C*), electron transfer flavoprotein (*D*, *ETF*), and succinate dehydrogenase (*E*, *SDH*). Structures are also provided for target subunits containing flavin cofactors, indicating the location of the flavin (*yellow*) and relevant cysteine residues. *F*, structure of mitochondrial complex I, indicating subunits containing cysteine residues that are selectively modified by NO_2_^−^ during ischemia highlighted in *red* and the number of *S*-nitrosated cysteine residues given in *parentheses. G*, quantification of *S*-nitrosation status of complex I cysteine residues sensitive to modification by ischemic NO_2_^−^. *H*, structures of the flavin-containing NDUFV1 subunit, indicating the location of the flavin (*yellow*) and relevant cysteine residues. *I*, modification of the *S*-nitrosation status of cysteine residue 257 on AAC1 and AAC2 under different conditions.

To further explore how ischemic exposure to NO_2_^−^ might affect mitochondrial oxidoreductase function, we examined the localization of substantially *S*-nitrosated sites on these mitochondrial pathways. We focused specifically on mitochondrial oxidoreductase sites that were identified as a cluster that is selectively modified by ischemic NO_2_^−^ (supplemental Table S5). Within this cluster, we found 22 sites that mapped to subunits of the OXPHOS machinery (Fig. 6*A*) and many in regions that have been implicated in regulating oxidative metabolism. For example, complex IV is *S-*nitrosated on cysteine residues 112 or 115 (Fig. 6*B*), which co-ordinate Zn^2+^ binding and have been suggested to regulate aspects of complex function (33). Complex III is modified on cysteine residue 139 of subunit IV (Fig. 6*C*), proximal to the cytochrome *c* reduction site, whereas both the electron-transferring flavoprotein (Fig. 6*D*) and succinate dehydrogenase (Fig. 6*E*) are modified at locations proximal to their flavin oxidation sites. The OXPHOS complex containing the most ischemic *S*-nitrosation sites (8) was complex I, most notably on four cysteine residues on the NDUFV1 subunit containing the flavin mononucleotide that is the proximal electron acceptor from NADH (Fig. 6, *F–H*). In addition, there was substantial *S*-nitrosation on the ND3 subunit at cysteine 39, which is close to the ubiquinone binding site, and its *S*-nitrosation is established to inhibit complex I in the ischemic myocardium (16). Substantial *S*-nitrosation of cysteine residues on NDUFA10 and NDUFA11 was also observed (Fig. 6*G*). NDUFA11 contributes the contact points between complex I and complex III (34, 35), and the two cysteines may form two disulfides with a further two cysteines on NDUFA11 that were on peptides not observed by SNOxICAT.

In addition to ischemic NO_2_^−^ modifications that cluster to functional domains of OXPHOS complexes, it is noteworthy that, under basal conditions, substantial copper/ascorbate-sensitive modification was observed on conserved cysteine residues on two isoforms of the ADP/ATP carrier (AAC1 and AAC2). Both exhibited substantial copper/ascorbate-sensitive modification of cysteine 257 during normoxia (Fig. 6*I*), despite the two peptides containing cysteine 257 differing in sequence and, consequently, mass. Under basal conditions, this site was among the most substantially modified sites observed (60–100% *S*-nitrosated). The nature of this modification is unclear, as it was largely unmodified under ischemic conditions. This site may be of particular interest, as chemical modification of this cysteine residue has been shown to have substantial effects on AAC protein activity (36). Furthermore, chemical and redox modifications of carrier proteins at this conserved cysteine site have been shown to affect carrier function and mitochondrial metabolism (37–39).

### Conclusions

The mechanism by which NO_2_^−^ modulates cardiac function *in vivo* is not clear. To address this, we applied ICAT labeling methods (22, 24) to identify and quantify *S*-nitrosation occupancy of protein cysteine residues across the proteome. Application of this approach, which we term SNOxICAT, allowed us to identify and quantify the targets of *S*-nitrosation by NO_2_^−^ in the heart *in vivo* for the first time.

Interestingly, only during ischemia were targets modified in the heart by NO_2_^−^. This suggests that the conditions during ischemia enhance protein *S*-nitrosation and that oxygen and/or pH are an important constraint on how NO_2_^−^ interacts with metabolism. *S*-nitrosation was not confined to a particular cellular compartment and was distributed throughout the tissue. The NO_2_^−^ anion itself is membrane-impermeant and so does not enter mitochondria, suggesting that ischemic NO_2_^−^ leads to mitochondrial *S*-nitrosation because of the membrane permeation of its conjugate acid HNO_2_. Quantitative assessment of the mitochondrial targets of *S*-nitrosation revealed a number of interesting candidates for further studies to assess the functional consequences of these modifications and provide an important starting point to further assess how NO_2_^−^ exerts its effects on mitochondrial function. In summary, we have developed a new technical approach that provides important new information on how NO_2_^−^ and NO_2_^−^ may have their beneficial effects *in vivo*.

## Experimental procedures

### Mouse experiments

For *in vivo* heart procedures, an open-chest, *in situ* heart model was used as described previously (40). Briefly, male C57BL/6 mice (8–10 weeks old, Charles River Laboratories) were anesthetized with sodium pentobarbital (70 mg/kg intraperitoneally), intubated endotracheally, and ventilated at 110 breaths/min with a tidal volume between 125 and 150 ml. A thoracotomy was performed, and a prominent branch of the left anterior descending coronary artery was surrounded by a 7-0 Prolene suture that was passed through a small plastic tube. Ischemia was induced by tightening the tubing against the heart surface. Mice were divided into four groups: normoxia (sham operation in which the suture was placed but the left anterior descending coronary artery was not occluded), normoxia + 10 mg/kg systemic intravenous sodium nitrite, 30 min of ischemia, and 30 min of ischemia + 10 mg/kg systemic intravenous sodium nitrite administered at 25 min of ischemia (31).

### Cys-ICAT labeling of heart tissue

At all times during *S*-nitrosothiol derivatization, samples were shielded from light. Following *in vivo* interventions, heart tissue was rapidly excised and immediately homogenized in ice-cold 20% TCA containing 0.5% sulfanilamide. The homogenate was incubated on ice for 30 min and then pelleted for 30 min at 16,000 × *g* at 4 °C. The pellet was washed with 10% and 5% (w/v) TCA and then resuspended in 80 μl of denaturing alkylating buffer (6 m urea, 2% (w/v) SDS, 200 mm Tris-HCl, 10 mm EDTA, 100 μm diethylenetriamine pentaacetate (DTPA), and 10 μm neocuproine). The contents of one vial of cysteine-reactive cleavable ICAT reagent (AB SCIEX) was added to each biological replicate sample to label reduced cysteine residues at 37 °C and mixed at 1400 rpm for 2 h on an Eppendorf Thermomixer. Sample protein was precipitated with 20% TCA, incubated at 4 °C for 30 min, and pelleted at 4 °C and 16,000 × *g* for 30 min. The pellet was washed twice as above and then resolubilized in 100 μl of denaturing alkylating buffer containing 10 μm CuSO_4_ and 1 mm ascorbic acid to reduce protein *S*-nitrosothiols in the presence of a second isotopically heavy, cysteine-reactive, cleavable ICAT reagent. Proteins were incubated at 37 °C and mixed at 1400 rpm for 2 h. The amount of protein to be processed was optimized to ensure saturation of thiol labeling by the ICAT reagent according to the instructions of the manufacturer. The sample was precipitated with 5 volumes of ice-cold acetone, stored at −20 °C for 2 h, and pelleted at 4 °C at 16,000 × *g* for 30 min. The pellet was washed twice with 90% ice-cold acetone and resuspended in denaturing buffer, and then 1 volume of trypsin (AB SCIEX, resuspended in H_2_O) was added (both provided by the ICAT kit). Proteins were digested at 37 °C with mixing at 1400 rpm overnight. Digested peptides were enriched for cysteine-containing peptides first on a cation exchange cartridge and then on an avidin affinity cartridge (AB SCIEX) as described previously (24). Eluted cysteine-containing, ICAT-labeled peptides were dried down overnight in a SpeedVac, and the biotin moiety of the ICAT label was removed by incubation with the cleaving reagents provided in the ICAT kit for 2 h at 37 °C with mixing at 1400 rpm). The isolated peptides were then dried down in a SpeedVac for MS analysis.

### LC/MS-MS analysis of peptides and data analysis

The LC/MS-MS analysis of the SNOxICAT-labeled peptides was carried out using an Orbitrap LTQ XL (Thermo Scientific), where peptides were analyzed after chromatography on a nanoscale reverse-phase column (75-μm inner diameter, 100-mm length) with a gradient of 0–40% buffer B (95% acetonitrile and 0.1% formic acid) in buffer A (5% acetonitrile and 0.1% formic acid) over 84 min at 300 nl/min. Four biological replicates were processed for each experiment. Peptides were identified using Proteome Discoverer (Thermo Scientific) and the Mascot protein identification program (Matrix Science). All experiments used the Mouse UniProt database (2013_12). Searches were performed using a 5-ppm precursor ion tolerance, requiring the N/C terminus of each peptide to have trypsin protease specificity and allowing up to two missed cleavages. ICAT tags on cysteine residues (+227.13 and +236.16 Da) and methionine oxidation (+15.99 Da) were set as variable modifications. Because many peptides were not observed in all replicates, the raw files were then analyzed using MassChroQ software, which quantifies peaks lacking MS2 sequence information by aligning them using the retention time of peptides common to runs for which MS2 information was obtained (30). An *in silico* tryptic digest of the complete mouse proteome was performed using a modified proteogest script generating a file that listed all cysteine-containing tryptic peptides with none, one, or two missed cleavages, the cysteine residue number, and the Uniprot accession number of the protein (41). The proteogest script modification was done by Dr. Alan Robinson (Medical Research Council Mitochondrial Biology Unit). The peptide list generated by proteogest was used together with the output from MassChroQ to sum the intensity from all peptide peak signals containing the cysteine residue of interest (*i.e.* miss-cleaved, methionine oxidation, different *z* values) and give a combined signal for both the heavy and light forms of the peptide. A significant complication with quantifying *S*-nitrosation compared with general oxidation is that many cysteines are not *S-*nitrosated at all, and consequently, in this study under control conditions, ∼55% of residues in a particular replicate have only one ICAT label. A failure to observe both labels can also occur for technical reasons related to MS instrumentation and software, or it can arise because the residue is either not *S-*nitrosated at all or it is completely *S-*nitrosated. The simplest mechanism for resolving this is to discard all peptides that lack both peaks, but excluding 55% of the dataset would give a misleading picture of *in vivo S-*nitrosation. Conversely, treating a failure of the instrumentation and software to find the correct peak as a biological result is also misleading.

To differentiate between these two situations and provide a more accurate representation of *in vivo S*-nitrosation, we utilized the expected similarity of informative data points across the four biological replicates. All data points with an S.E. of less than 6 were considered informative and automatically included in the subsequent analyses, comprising 78% of the total. For data points with an S.E. ≥ 6, heavy and light ICAT signals were separately cross-referenced to the median heavy or light intensity of that cysteine in the four biological replicates. Those with an intensity signal ≤20% of the heavy or light median intensity were excluded. This step prevents a single technical failure with either a heavy or light peak in one replicate, causing a group of four replicates to be eliminated from analysis. This was necessary, as only 387 cysteine residues (∼20%) were sufficiently *S-*nitrosated to be observed with both heavy and light isotope labels. Finally, all data points that still had an S.E. ≥ 8 or *n* < 3 were excluded. *S*-nitrosation is presented as the percentage (100 × HeavyICAT) / (HeavyICAT + LightICAT).

Information on the subcellular localization of the proteins identified and the biological processes they participated in was obtained using MitoMiner (http://mitominer.mrc-mbu.cam.ac.uk/)^5^ (42). For bar charts, the data for distribution of proportion of cysteine residues against percentage *S*-nitrosation were generated by counting the number of cysteine residues in each 2% quantile for each individual biological replicate and then averaging the relative values for each quantile over the four biological replicates. For volcano plots of the extent of *S-*nitrosation of a given cysteine under one condition against its *S-*nitrosation under another condition, the significance of the shift in *S-*nitrosation was assessed by an *F* test for variance followed by the appropriate two-tailed Student's *t* test, assuming equal or unequal variance as appropriate. A Benjamini-Hochberg procedure was used to correct for multiple comparisons and thus determine significant changes.

### Mitochondrial experiments

Liver mitochondria were prepared from female Wistar rats killed by stunning and cervical dislocation, followed by homogenization of the liver and differential centrifugation in ice-cold STE buffer (250 mm sucrose, 10 mm Tris, and 1 mm EGTA (pH 7.4)). The protein concentration was determined by the biuret assay using bovine serum albumin as a standard. For swelling experiments, mitochondria (0.5 mg protein/ml) were incubated in 10 mm TrisHCl (pH 7.2) at 37 °C supplemented with rotenone (4 μg/ml) and containing either KNO_2_ or KNO_3_ (100 mm). Absorbance was measured at 550 nm, and valinomycin (1 μm) or FCCP (2 μm) was added where indicated. Fig. 5*E* shows a typical result of three replicates.

## Author contributions

C. M. and V. R. P. carried out the mouse experiments, supervised by T. K. K. E. M., E. T. C., and I. M. F. established the ICAT protocol and carried out the mass spectrometry. E. T. C. and A. M. J. carried out the data analysis. B. K. E. performed the bioinformatics analyses. T. A. P., T. P. B., M. F., and G. Y. L. carried out experiments on isolated mitochondria. E. T. C., A. M. J., T. K., and M. P. M. directed the project and wrote the manuscript.

## Supplementary Material

Supplemental Data

## Footnotes

5 Please note that the JBC is not responsible for the long-term archiving and maintenance of this site or any other third party–hosted site.

## Footnotes

4 The abbreviations used are:

ICAT: isotope-coded affinity tag
FCCP: carbonyl cyanide *p*-trifluoromethoxyphenylhydrazone
AAC: ADP/ATP carrier
SNOxICAT: *S*-nitrosothiol redox isotope-coded affinity tag
OxICAT: redox isotope-coded affinity tag
OXPHOS: oxidative phosphorylation.
