# Supplementary material for: Identification and quantification of protein S-nitrosation by nitrite in the mouse heart during ischemia
Source: J Biol Chem. 2017 Jul 14;292(35):14486–95. doi: 10.1074/jbc.M117.798744 (PMC5582841; doi:10.1074/jbc.M117.798744)
Supplement: Supplemental Data [file supp_292_35_14486__index.html]

Identification and Quantification of Protein S-nitrosation by Nitrite in the Mouse Heart during Ischemia — Identification and quantification of protein S-nitrosation by nitrite in the mouse heart during ischemia — Measuring cardiac protein S-nitrosation — Supplemental Data 

# Identification and quantification of protein *S*-nitrosation by nitrite in the mouse heart during ischemia

## Supplemental Data

- Supplementray information (.pdf, 952 KB) - Sup figures
- Sup table 1 (.xlsx, 724 KB) - Sup table 1
- Sup Table 2 (.xlsx, 538 KB) - Sup Table 2
- Sup Table 3 (.xlsx, 610 KB) - Sup Table 3
- Sup Table 4 (.xlsx, 37 KB) - Sup Table 4
- Sup Table 5 (.xlsx, 130 KB) - Sup Table 5
