# Supplementary material for: Identification and quantification of protein S-nitrosation by nitrite in the mouse heart during ischemia
Source: J Biol Chem. 2017 Jul 14;292(35):14486–95. doi: 10.1074/jbc.M117.798744 (PMC5582841; doi:10.1074/jbc.M117.798744)
Supplement: Supplemental Data [file 10.1074_M117.798744_jbc.M117.798744-1.pdf]

SUPPLEMENTARY INFORMATION

Identification and Quantification of Protein *S*-nitrosation by Nitrite in the Mouse Heart during Ischemia

Edward T. Chouchani[a,b*], Andrew M. James[c,*], Carmen Methner[d], Victoria R. Pell[d], Tracy A. Prime[c], Brian K. Erickson[a,b], Marleen Forkink[c], Gigi Y. Lau[c], Thomas P. Bright[c], Katja E. Menger[c], Ian M. Fearnley[c], Thomas Krieg[d] and Michael P. Murphy[c,*]

From the [a]Department of Cancer Biology, Dana–Farber Cancer Institute, Boston, MA, USA.
[b]Department of Cell Biology, Harvard Medical School, Boston, MA, USA
[c]MRC Mitochondrial Biology Unit, University of Cambridge, Cambridge Biomedical Campus, CB2 0XY, UK.
[d]Department of Medicine, University of Cambridge, Addenbrooke's Hospital, Hills Road, Cambridge, CB2 2QQ, UK.

Figure S1

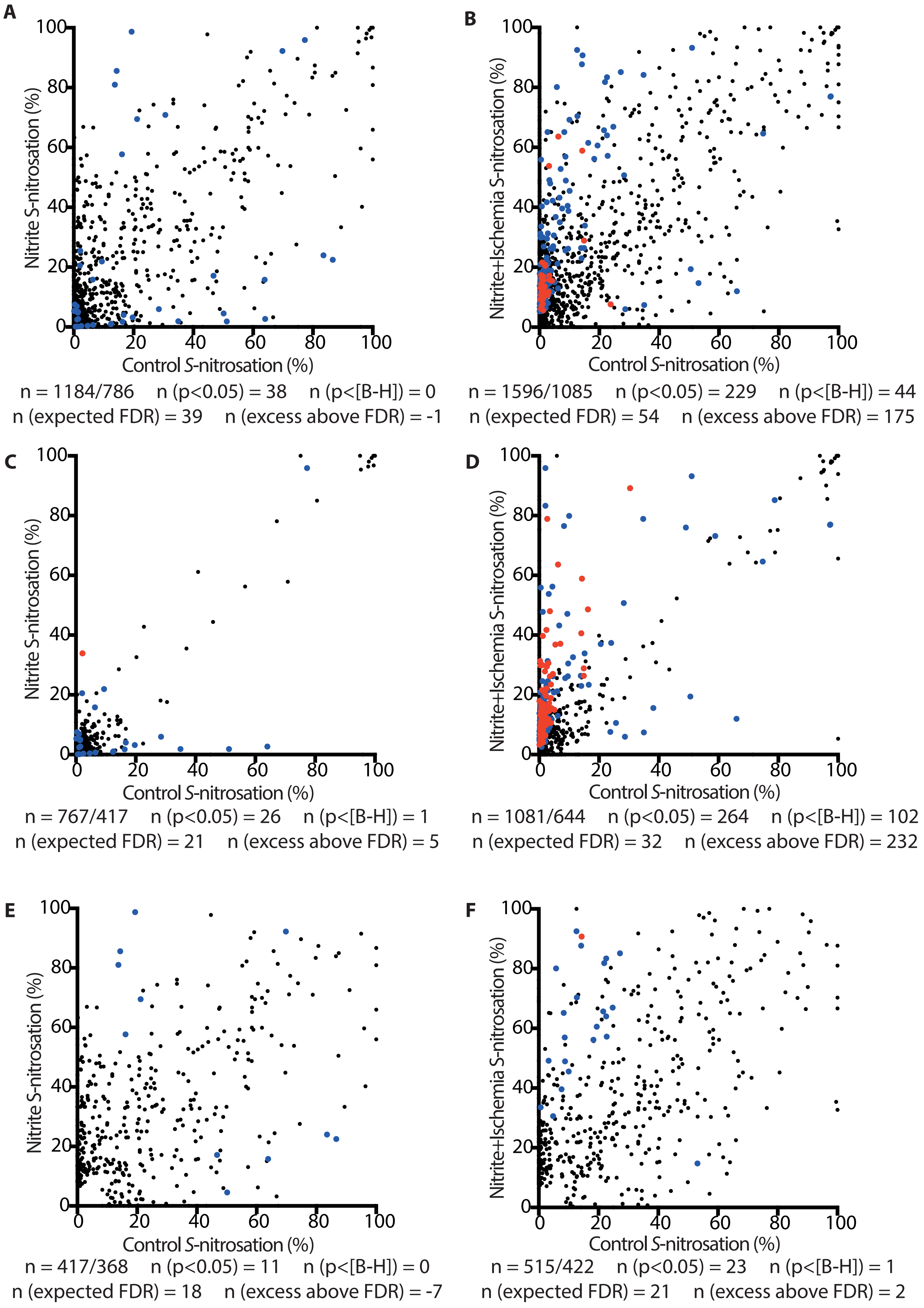
A
Nitrite S-nitrosation (%)
Control S-nitrosation (%)
n = 1184/786 n (p<0.05) = 38 n (p<[B-H]) = 0
n (expected FDR) = 39 n (excess above FDR) = -1
B
Nitrite+Ischemia S-nitrosation (%)
Control S-nitrosation (%)
n = 1596/1085 n (p<0.05) = 229 n (p<[B-H]) = 44
n (expected FDR) = 54 n (excess above FDR) = 175
C
Nitrite S-nitrosation (%)
Control S-nitrosation (%)
n = 767/417 n (p<0.05) = 26 n (p<[B-H]) = 1
n (expected FDR) = 21 n (excess above FDR) = 5
D
Nitrite+Ischemia S-nitrosation (%)
Control S-nitrosation (%)
n = 1081/644 n (p<0.05) = 264 n (p<[B-H]) = 102
n (expected FDR) = 32 n (excess above FDR) = 232
E
Nitrite S-nitrosation (%)
Control S-nitrosation (%)
n = 417/368 n (p<0.05) = 11 n (p<[B-H]) = 0
n (expected FDR) = 18 n (excess above FDR) = -7
F
Nitrite+Ischemia S-nitrosation (%)
Control S-nitrosation (%)
n = 515/422 n (p<0.05) = 23 n (p<[B-H]) = 1
n (expected FDR) = 21 n (excess above FDR) = 2

FIGURE S1: Effects of dataset curation. (A) Non-curated *S*-nitrosation of normoxic control and nitrite-treated heart *in vivo*. (B) Non-curated *S*-nitrosation of normoxic control and ischemic nitrite-treated heart *in vivo*. (C) Curated *S*-nitrosation of normoxic control and nitrite-treated heart *in vivo*. (D) Curated *S*-nitrosation of normoxic control and ischemic nitrite-treated heart *in vivo*. (E) Excluded *S*-nitrosation values from normoxic control and nitrite-treated heart *in vivo*. (F) Excluded *S*-nitrosation values from normoxic control and ischemic nitrite-treated heart *in vivo*. n = number of data points (black)/number of statistical comparisons. n ($p < 0.05$) = number of data points (blue) for which there is a statistical difference by the Student's t-test with no correction for multiple comparisons. n (p < B-H) = number of data points (red) for which there is a statistical difference when corrected for multiple comparisons by the Benjamini-Hochberg procedure. n (expected FDR) = number of falsely significant data points expected for a dataset of this size. n (excess above FDR) = number significant data points more than expected from the FDR for a dataset of this size.

Supplementary Table Legends

TABLE S1: Summed HeavyICAT or LightICAT *S*-nitrosation intensity values in *in vivo* mouse heart. The minimal tryptic peptide is the cysteine-containing peptide present in the database and not necessarily the peptide sequence of the extracted-ion chromatogram (XIC) detected by LC-MS/MS. Data are the summed heavyICAT and lightICAT intensity from all the XICs belonging to each cysteine residue observed. Mitochondrial localization was assigned by IMPI.

TABLE S2: Raw *S*-nitrosation occupancy (%) in in vivo mouse heart. Data is expressed as ((100 x HeavyICAT)/(HeavyICAT+LightICAT)) using data from Table S1. The minimal tryptic peptide is the cysteine-containing peptide present in the database and not necessarily the peptide sequence of the extracted-ion chromatogram (XIC) detected by LC-MS/MS. Mitochondrial localization was assigned by IMPI.

TABLE S3: Curated *S*-nitrosation occupancy (%) in in vivo mouse heart. The minimal tryptic peptide is the cysteine-containing peptide present in the database and not necessarily the peptide sequence of the extracted-ion chromatogram (XIC) detected by LC-MS/MS. Values for each cysteine residue from Table S2 were curated if *S*-nitrosation SEM > 6. Individual biological replicates were excluded if HeavyICAT or LightICAT intensity from Table S1 < 20% of the median for the 4 biological replicates. Average values are only calculated if the cysteine residue was observed at least 3 times after curation in a particular condition. Finally data points where cysteine oxidation is variable (SEM > 8%) were excluded. Data is expressed as (100 x HeavyICAT)/(HeavyICAT+LightICAT).

TABLE S4: Cysteine residues *S*-nitrosated in response to NO_2^- in the ischemic mouse heart. The minimal tryptic peptide is the cysteine-containing peptide present in the database and not necessarily the peptide sequence of the extracted-ion chromatogram (XIC) detected by LC-MS/MS. Data is expressed as (100 x HeavyICAT)/(HeavyICAT+LightICAT) in ischemic mouse heart exposed to NO_2^- minus (100 x HeavyICAT)/(HeavyICAT+LightICAT) from either control hearts or those exposed to NO_2^- or ischemia alone. Significance was calculated using an f-test for equal variance followed by the appropriate Students t-test. The Benjamini-Hochberg procedure was used to correct for multiple comparisons and p values are displayed if this procedure deemed them significant. Many have no pvalue because one of the conditions lacked variance.

TABLE S5: Pathway analyses of protein cysteine residues that are substantially (>10%) differentially *S*-nitrosated between conditions.
